# Seroprevalence of viral and bacterial diseases among the bovines in Himachal Pradesh, India

**DOI:** 10.14202/vetworld.2017.1421-1426

**Published:** 2017-12-03

**Authors:** Shailja Katoch, Shweta Dohru, Mandeep Sharma, Vikram Vashist, Rajesh Chahota, Prasenjit Dhar, Aneesh Thakur, Subhash Verma

**Affiliations:** 1 Department of Veterinary Microbiology, Dr. G.C. Negi College of Veterinary & Animal Sciences, CSK Himachal Pradesh Agricultural University, Palampur - 176 062, Himachal Pradesh, India; 2 Department of Animal Husbandry, Government of Himachal Pradesh, Himachal Pradesh, India

**Keywords:** bacterial diseases, seroprevalence, viral diseases

## Abstract

**Aim:**

The study was designed to measure the seroprevalence of viral and bacterial diseases: Infectious bovine rhinotracheitis, bovine viral diarrhea, bovine leukemia, bovine parainfluenza, bovine respiratory syncytial disease, brucellosis, and paratuberculosis among bovine of Himachal Pradesh during the year 2013-2015.

**Materials and Methods:**

The serum samples were collected from seven districts of state, namely, Bilaspur, Kangra, Kinnaur, Lahul and Spiti, Mandi, Sirmour, and Solan. The samples were screened using indirect ELISA kits to measure the seroprevalence of viral and bacterial diseases.

**Results:**

The overall seroprevalence of infectious bovine rhinotracheitis was 24.24%, bovine viral diarrhea 1.52%, bovine leukemia 9.09%, bovine parainfluenza 57.58%, bovine respiratory syncytial disease 50%, brucellosis 19.69%, and paratuberculosis 9.09% in Himachal Pradesh. The seroprevalence of bovine rhinotracheitis, bovine leukemia, bovine parainfluenza, bovine respiratory syncytial disease, and paratuberculosis in the state varied significantly (p<0.01) while was insignificant for bovine viral diarrhea and brucellosis (p>0.01). Multiple seropositivity has been observed in this study. Bovine parainfluenza virus 3 was observed commonly in mixed infection with almost all viruses and bacteria under study.

**Conclusion:**

The viral and bacterial diseases are prevalent in the seven districts of Himachal Pradesh investigated in the study. Therefore, appropriate management practices and routine vaccination programs should be adopted to reduce the prevalence of these diseases.

## Introduction

Bovine contribute significantly to the economy of marginal farmers, especially those living in the mountainous and the semi-mountainous regions of Himachal Pradesh through milk, milk products, skin, manure, etc. The advancement in animal husbandry practices including animal health has greatly helped in improving the socioeconomic status of the rural population in the state. Despite all this, infectious diseases continue to considerably decrease the livestock production and hamper the livestock economy in the state.

The viral diseases: Infectious bovine rhinotracheitis, bovine viral diarrhea, bovine leukemia, bovine parainfluenza, and bovine respiratory syncytial disease are caused by bovine herpesvirus type 1 (BHV-1), bovine viral diarrhea virus (BVDV), bovine leukemia virus (BLV), bovine parainfluenza type 3 virus (BPI-3V), and bovine respiratory syncytial virus (BRSV), respectively. The bacterial diseases: Brucellosis and paratuberculosis are caused by *Brucella* spp. and *Mycobacterium avium* subsp. *Paratuberculosis*, respectively. All these diseases contribute to both direct and indirect economic losses. Direct loss is value of the animal that dies of disease whereas indirect losses include production losses which include decreased milk yield and reduced draught power and impairment of reproductive potential of animals.

Information on prevalence of these diseases is available from a number of states in which these diseases has been reported in India. However, only few reports on prevalence of some of the diseases, namely, brucellosis, infectious bovine rhinotracheitis, and bovine leukemia are available from Himachal Pradesh [1-3] so far. No information on the status of other diseases in present. Efficient and sensitive diagnostic tests are very useful in providing quick evidence indicating presence or absence of the disease.

Therefore, the current study was performed to generate baseline data to determine the prevalence of important viral and bacterial diseases in the state using indirect ELISA which could help in devising preventive and control strategies against these diseases.

## Materials and Methods

### Ethical approval

The study was approved by Institute Animal Ethics Committee. The blood samples were collected from the animals for serum extraction only after consent of the owners. The ethical considerations in accordance to Institute Animal Ethics Committee related to animal handling were observed to ensure no discomfort or pain to animal during sampling.

### Animals and their housing

The study was conducted during the period of 2013-2015, on crossbred (Jersey X Red Sindhi or Jersey X local Pahari) adult cattle from different districts of Himachal Pradesh. They were housed in loose housing system in State Government cattle farms of Kothipura (Bilaspur), Palampur (Kangra), and Bagthan (Sirmour) and in conventional barn system in Mandi, Solan, Kinnaur, and Lahul and Spiti district. The animals were given proper feed and were housed following appropriate husbandry and good management practices. All the animals under the study were vaccinated for Foot and Mouth Disease, Hemorrhagic Septicemia, and Black quarter disease as per the vaccination schedule of the Department of Animal Husbandry, Himachal Pradesh [4]. In Himachal Pradesh, no vaccination is being practiced against the diseases discussed in this study [4].

### Samples and sampling

A total of 132 serum samples were collected from apparently healthy bovines randomly with no history of illness past 1 month for diagnosis of infectious bovine rhinotracheitis, bovine viral diarrhea, bovine leukemia, bovine parainfluenza, bovine respiratory syncytial disease, paratuberculosis, and brucellosis. The samples were collected from seven different districts: Bilaspur, Kangra, Kinnaur, Lahul and Spiti, Mandi, Sirmour, and Solan of Himachal Pradesh (Table-1). All of the seven districts are located in different terrains and agro-climatic zones of the state resulting in a random distribution. For serum, 5 ml whole blood was collected through jugular venipuncture in a vacutainer (BD vacutainer) and then allowed to clot at room temperature for 20 min. The serum was separated, clarified by centrifugation at 3000 rpm for 10 min and stored at −20°C till the tests were performed.

**Table-1:** Seroprevalence of viral and bacterial diseases in Himachal Pradesh.

District	Number of samples	Seropositive for BHV-1 (%)	Seropositive for BVDV (%)	Seropositive for BLV (%7)	Seropositive for BPI-3V (%)	Seropositive for BRSV (%)	Seropositive for brucellosis (%)	Seropositive for paratuberculosis (%)
Sirmaur	20	6 (30)	0 (0)	0 (0)	6 (30)	20 (100)	2 (10)	0 (0)
Solan	18	2 (11.11)	0 (0)	0 (0)	2 (11.11)	0 (0)	6 (33.33)	2 (11.11)
Kangra	18	0 (0)	0 (0)	0 (0)	18 (100)	18 (100)	4 (22.22)	6 (33.33)
Lahaul and Spiti	20	18 (90)	2 (10)	0 (0)	16 (80)	4 (20)	2 (10)	0 (0)
Bilaspur	18	0 (0)	0 (0)	2 (11.11)	18 (100)	14 (77.78)	6 (33.33)	0 (0)
Mandi	18	2 (11.11)	0 (0)	10 (55.56)	6 (33.33)	8 (44.44)	4 (22.22)	0 (0)
Kinnaur	20	4 (20)	0 (0)	0 (0)	10 (50)	2 (10)	2 (10)	4 (20)
Total	132	32 (24.24)	2 (1.52)	12 (9.09)	76 (57.58)	66 (50)	26 (19.69)	12 (9.9)

BHV-1=Bovine herpes virus type 1, BVDV=Bovine viral diarrhea virus, BLV=Bovine leukemia virus, BPI-3V=Bovine parainfluenza type 3 virus, BRSV=Bovine respiratory syncytial virus

### Indirect ELISA

The indirect ELISA was done using Svanovir antibody test kits (BR Biochem Life Sciences, Delhi, India) for viral and bacterial diseases. The protocol for indirect ELISA was followed as per manufacturer’s guidelines.

### Statistical analysis

Estimation of seroprevalence of diseases with a 95% confidence interval (CI) and a Chi-square test were performed and data analysis was performed statistically using GraphPad Prism 5 software.

## Result and Discussion

The viral and bacterial diseases of bovines studied in the present study are major health problem of cattle worldwide and are important because they cause an increase in morbidity and mortality rates leading to economic losses. Since the animals were not vaccinated with any of the aforementioned diseases in the current study, the results may indicate that these viruses and bacteria are circulating within the animal populations in these areas. However, the present study only limited to detection of antibodies against these bacterial and viral pathogens and no tests were carried out to detect the pathogens as this was a preliminary study.

A total of 132 serum samples were screened from seven districts of Himachal Pradesh. The district wise sample collection and the overall and district wise seroprevalence of diseases is given in Table-1.

Infectious bovine rhinotracheitis is one among the key viral infections of the bovines and has been known to exist in India since 1976 [5]. Seroprevalence of infectious bovine rhinotracheitis has been reported from different states of India [6,7]. Overall, nationwide prevalence rate of disease was found around 34% with differing prevalence rates reported by various studies from different parts: 39% from north-eastern, 37% northern, 25% central, 24% western, and 17% eastern region [8,9]. Previously in Himachal Pradesh, 50% seroprevalence of BHV-1 was reported using avidin biotin-ELISA [2]. However, in this study, the prevalence of the disease was 24.24% (CI=−1.25-10.39). The seroprevalence of BHV-1 varied significantly (Chi-square=62.55, p<0.01) from a low of 0% to a high of 90% in various districts of the state. The seroprevalence of BHV-1 in the present study showed considerable reduction when compared to earlier study [2]. There could be different reasons for this with one plausible explanation could be owed to increased penetration of artificial insemination in the state with semen coming from bulls free of BHV-1 infections. The prevalence of BHV-1 was recorded highest in the tribal district of Lahul and Spiti; however, reasons for this could not be ascertained. As the information regarding the status of breeding (natural or artificial) was not available, it was not possible to ascertain the reason for high prevalence in this district. One possibility for high infection rate could be use of bulls for natural breeding in cattle instead of artificial insemination. As there is no vaccination schedule for BHV-1 in the state the seropositivity only reflect exposure to virus. The animal with BHV-1 seropositivity may reflect the proportion of carrier animals as the virus remains in latent form in nerve ganglions and is resecreted during immunosuppression in the animals caused by corticosteroid injections, or due to stress imposed in the animal during transportation, calving, overcrowding, etc.

BVDV has been reported to be the virus most commonly isolated from pneumonic lungs of cattle. The seroprevalence of BVDV was recorded 15.3% in cattle and 23.2% in buffalo of 16 and 9 states of India, respectively [10], while a prevalence of 24.7% was reported recently from smallholder dairy units of India [11]. In an earlier study [10], no samples were collected from the Himachal Pradesh and the serum samples collected from cattle of neighboring states, namely, Jammu and Kashmir, Punjab, and Haryana were also found to be negative. In this study, very low seropositivity of BVDV was observed as only 1.52% (CI=−0.41-0.98) of cattle serum harbored antibodies to this virus in Himachal Pradesh. The seroprevalence of BVD is not significant (Chi-square=11.37, p>0.01). The seronegativity for the virus in the neighboring states may be one of the reasons for very low seropositivity for BVDV in Himachal Pradesh as well.

In India, the status of BLV infection in crossbred zebu cattle was first reported in two cattle herds of the country [12]. The seroprevalence of BLV was highly variable in crossbred zebu cattle of nine states of India and was in the range of 5.4-75% [1], and in case of Himachal Pradesh; 40.62% animals carried antibodies against this virus. However, in this study, the seroprevalence of BLV was low and stood at and is 9.09% (CI=−1.73-5.16) in Himachal Pradesh. The seroprevalence of BLV varied significantly (Chi-square=56.71, p<0.01) from a low of 0% to a high of 55.56% in various districts of the state. The wide difference between the results of these two studies could possibly due to different areas surveyed. It is evident from this study as well where out of seven districts surveyed five districts had no seropositivity in animals screened; however, the animals from the other two districts showed the prevalence of 33.33%.

In cattle, respiratory infections cause serious economic losses. BPI-3V alone does not produce any serious disease but with other respiratory viruses such as BRSV and BHV-1 it can lead to serious complications due to immunosuppression induced by BPI-3V [13]. The virus in association with BRSV and BHV-1 causes respiratory disease complex in cattle. The seroprevalence of BPI-3V was reported 20% in the cattle from dairy farm of Punjab [14]. In this study, the seroprevalence of BPI-3V has been recorded at 57.58% (CI=4.83-16.88) in Himachal Pradesh. The seroprevalence of BPI-3V varied significantly (Chi-square=57.58, p<0.01) from a low of 11.11% to a high of 100% in various districts of the state. The difference in weather conditions [15] and the ubiquitous nature of virus [16] is responsible for variation in the seroprevalence of the virus. The higher seroprevalence of BPI-3V indicates the presence of respiratory infection among the cattle in Himachal Pradesh as it acts as a predisposing factor for the respiratory disease complex.

BRSV effect cattle of all age groups but is the major cause of respiratory diseases in the calves and also leads to severe economic losses [17]. Seroprevalence of BRSV infection in cattle from Orissa was 46.1% by indirect ELISA and 65.3% by sandwich ELISA [18] and 47.05% in Punjab by competitive ELISA [19]. Similar to this, seropositivity of BRSV in Himachal Pradesh in the present study was observed in 50% animals CI (2.05-16.81). The seroprevalence of BRSV varied significantly (Chi-square=81.78, p<0.01) from a low of 0% to a high of 100% in various districts of the state. The higher seroprevalence of BPI-3V, BRSV, and BHV-1 among cattle of Himachal Pradesh indicates exposure to these viruses as the vaccination is not practiced in the state against these diseases.

Brucellosis is one of the five major notifiable bacterial diseases of zoonotic importance in the world. Brucellosis is a disease of animals with humans as an accidental host [20]. Seroprevalence of brucellosis has been reported from different states of India [21-23]. The overall prevalence rate of antibodies was 1.9% in cattle and 1.8% in buffalo [21]. Progress reports of monitoring programs from 2012 to 2013 by the Indian Council of Agricultural Research estimate that the current national seroprevalence of brucellosis in cattle is roughly 13.5% and at a stable, endemic equilibrium [24]. In this study, the seroprevalence of brucellosis was found to be 19.69% (CI=2.05-5.37) in Himachal Pradesh. The seroprevalence of brucellosis is not significant (Chi-square=81.78, p>0.01). There is an increase in the seroprevalence of brucellosis in the state as in earlier studies [3,25] it was reported to be 11.08% and 0.3%, respectively. The reasons which might have contributed to increased seroprevalence are the ban on slaughter of known positive cases due to socioeconomic conditions of India, changing husbandry practices of rearing of animals, overstocking of animals [25], unrestricted trade and movement of animals [26], free grazing and mixing with flocks of sheep and goats, use of common pastures and water sources. Another important factor contributing to increased seroprevalence is free movement of animals practiced by the gaddis, gujjars, and bakarwals, who own about 70% of migratory sheep and goat population [27]. The present study revealed that there was an increase in seroprevalence of the disease in the state which is a serious threat to both human and animal health. Therefore, it requires a constant monitoring system to study the disease dynamics so that the control strategies can be implemented to lower down the prevalence of the disease in the state.

Paratuberculosis is a chronic debilitating disease of ruminants and is one among the major diseases of ruminants worldwide. High prevalence of JD [28] with low systematic monitoring of the same makes it more significant in India. The seroprevalence of paratuberculosis in a selected population of cattle from different parts of India was 21.3% [29]. Similarly, 22.5% and 20% seroprevalence of paratuberculosis in indigenous and crossbred cattle, respectively, was reported previously from different parts of India [30]. High seroprevalence of paratuberculosis average 29% (29.8% in cattle and 28.6% in buffalo) has been found in domestic animals using indigenous, sensitive and MAP-specific ELISA kits in North India. The seroprevalence of paratuberculosis in Uttar Pradesh (31.9%), Punjab (23.3%), Gujarat (13.39%), and Andhra Pradesh (16.26%) has been reported [29-32]. In Himachal Pradesh, the seroprevalence of the disease was reported 9.09% (CI=−0.5331-3.96) in this study. The seroprevalence of paratuberculosis varied significantly (Chi-square=23.37, p<0.01) from a low of 0% to a high of 100% in various districts of the state. The differences observed in the seroprevalence rate of diseases may be either due to different rates of exposure of the animal to the pathogen, various agro-climatic zones, or management practices followed in a particular region.

Among all the disease studied, the high prevalence of BPI-3V and BRSV antibodies were detected in 57.58% and 50.0% cattle, respectively. This indicates that most animals are exposed mainly to these two viruses. BPI3V and BRSV were more frequently associated together 8.33% (11/132). BPI3V was observed commonly in mixed infection with almost all viruses and bacteria under study. It has been suggested that seropositivity for BVDV/BPI-3V may indicate persistent infection and may serve as a risk of infection to non-immunized healthy animals [33]. According to our knowledge, this is the first report about detection of antibodies against BPI-3V, BVDV, BRSV, BLV, and paratuberculosis infections in Himachal Pradesh, India.

The seroprevalence data were also evaluated in terms of single and multiple seropositivity. 28.79% (38/132) of animal were seropositive for a single disease. 15.91% (21/132), 12.12% (16/132), and 0.76% (1/132) of animal were seropositive for two, three, and four diseases, respectively. One animal was seropositive for each (BPI3V + Paratuberculosis), (BPI3V + Paratuberculosis + Brucellosis), (BPI3V + BRSV + BLV), (BPI3V + BVDV + BHV1), (BLV + BHV1 + Brucella) and (BPI3V + BRSV + BHV1 + Brucella), two each for (BRSV + BHV1) and (BHV1 + Brucella), three each for (BPIV3 + BRVS + Paratuberculosis) and (BPI3V + BRSV + BHV1), five animals for (BPI3V + BHV1), six animals for (BPI3V + BRSV + Brucella), and eleven were positive for (BPI3V + BRSV). Multiple seropositivity has been observed in this study. Similar multiple seropositivity has been observed earlier [14,34].

## Conclusion

The present study revealed the seroprevalence of infectious bovine rhinotracheitis, bovine viral diarrhea, bovine leukemia, bovine parainfluenza, bovine respiratory syncytial disease, brucellosis, and paratuberculosis in Himachal Pradesh. These preliminary observations should be further followed up by studies for the isolation of etiological agents of these diseases from seropositive animals to establish whether these diseases exist in Himachal Pradesh. In addition, studies to evaluate the impact of these diseases on the animal health and production should be carried out. Use of appropriate management practices and routine vaccination programs may reduce the incidence of these diseases.

## Authors’ Contributions

SK, SD, and SV carried out the experiments. SK collected the scientific literature and drafted the manuscript. SV, VV, RC, PD, and AT helped in collection of serum samples from different districts of the state. SV guided during planning of experiments and final draft of manuscript. RC, PD, AT, and VV revised the manuscript. All authors read and approved the final manuscript.
